# Combined deletion of MEN1, ATRX and PTEN triggers development of high-grade pancreatic neuroendocrine tumors in mice

**DOI:** 10.1038/s41598-024-58874-2

**Published:** 2024-04-12

**Authors:** Mary Esmeralda Fuentes, Xiaoyin Lu, Natasha M. Flores, Simone Hausmann, Pawel K. Mazur

**Affiliations:** 1 Department of Experimental Radiation Oncology, The University of Texas MD Anderson Cancer Center, Houston, TX 77030 USA; 2 The University of Texas MD Anderson Cancer Center UT Health Graduate School of Biomedical Sciences, Houston, TX 77030 USA

**Keywords:** Cancer, Cancer models

## Abstract

Pancreatic neuroendocrine tumors (PanNETs) are a heterogeneous group of tumors that exhibit an unpredictable and broad spectrum of clinical presentations and biological aggressiveness. Surgical resection is still the only curative therapeutic option for localized PanNET, but the majority of patients are diagnosed at an advanced and metastatic stage with limited therapeutic options. Key factors limiting the development of new therapeutics are the extensive heterogeneity of PanNETs and the lack of appropriate clinically relevant models. In that context, genomic sequencing of human PanNETs revealed recurrent mutations and structural alterations in several tumor suppressors. Here, we demonstrated that combined loss of MEN1, ATRX, and PTEN, tumor suppressors commonly mutated in human PanNETs, triggers the development of high-grade pancreatic neuroendocrine tumors in mice. Histopathological evaluation and gene expression analyses of the developed tumors confirm the presence of PanNET hallmarks and significant overlap in gene expression patterns found in human disease. Thus, we postulate that the presented novel genetically defined mouse model is the first clinically relevant immunocompetent high-grade PanNET mouse model.

## Introduction

Pancreatic neuroendocrine tumors (PanNETs) are a relatively rare neuroendocrine malignancy with an incidence of < 1 per 100,000 per year^1^ but account for approximately 2–5% of pancreatic malignancies and 6–7% of all neuroendocrine tumors and exhibit the most aggressive behavior among the latter. Although falsely considered benign neoplasia due to long periods of indolent growth, PanNETs are a heterogeneous group of tumors with often unpredictable and varying degrees of malignancy^2,3^. As many as 50–80% of PanNETs are associated with local and distal metastatic disease^4^. As a consequence of initially asymptomatic progression, the majority of patients are diagnosed at an advanced stage with limited therapeutic options. Thus, there is a critical unmet need for novel therapeutics and diagnostic modalities. Key factors limiting the development of new therapeutics are the extensive heterogeneity of PanNETs and the lack of appropriate clinically relevant in vivo models.

PanNETs are tumors that originate from endocrine cells within the islet of Langerhans^5^. These cells produce distinct hormones and are involved in many regulatory functions^6^. PanNETs that preserve hormone-producing attributes are called functional tumors (i.e. insulinomas, glucagonomas, somatostatinomas)^7^. Functional tumors often cause severe clinical syndromes, for example, insulinomas may lead to hypoglycemia^1,5^. In contrast, non-functional PanNETs do not secrete hormones and do not present with any specific clinical characteristics^3,7^. Despite these clinical differences, both functional and non-functional PanNETs can be aggressive, develop metastases, acquire treatment resistance, and have low overall survival rates^7^. The overall 5-year survival rate for PanNET patients is 53% with surgical resection. However, in patients with metastatic disease or for those unable to undergo surgery, the 5-year survival rate drastically drops to 23%^1^. For decades, surgery has remained the first line of defense^8^ and standard chemotherapeutics have been used in advanced patients with little to no avail^9,10^. Current targeted therapeutics include Sunitinib and Everolimus^11,12^, which show limited efficacy due to the development of acquired resistance^13^. Thus, there is a critical unmet need for translational tools to develop novel and effective precision therapeutics.

Recent sequencing efforts focused on elucidating the genomic landscape of PanNETs identified recurrent mutations and structural alterations in several tumor suppressors and chromatin modulators^14–16^. The most frequently mutated genes are *MEN1* (multiple endocrine neoplasia type 1), *ATRX* (alpha-thalassemia/mental retardation X-linked), *DAXX* (death-domain associated protein), and *PTEN* (phosphatase and tensin homolog). Mutations in MEN1 are commonly associated with neuroendocrine malignancies and have been studied before^17–23^. MENIN is a scaffold protein encoded by the MEN1 gene that functions in multiple biological processes controlling gene expression, DNA damage repair, cell proliferation, and motility. Those factions are mediated by MENIN interactions with a plethora of proteins^24,25^; most notably, MENIN is a component of a histone methyltransferase KMT2A complex that methylates lysine 4 of Histone H3 and functions as a transcriptional regulator^21,26^. Although MEN1 loss is commonly associated with pancreatic neuroendocrine tumorigenesis, the deletion of MEN1 in mouse pancreatic beta-cells results in a relatively modest phenotype, with indolent tumors developing with long latency^22^. The second and third most common mutations found in human PanNETs are found in ATRX and DAXX, which together form a histone chaperone complex that deposits histone variant H3.3 into repetitive heterochromatin and telomeric regions of the genome^27,28^. Mutations in the ATRX-DAXX complex are found in a variety of tumors, including adult lower-grade gliomas, pediatric glioblastoma multiforme, pediatric adrenocortical carcinoma, osteosarcoma, and neuroblastoma^28^. Deletion of DAXX or ATRX in pancreatic neuroendocrine cells fails to trigger neoplastic transformation^29–32^. Additional mutations found commonly in human PanNETs target mTOR pathway genes, including PTEN, TSC2, and PIK3CA^14,33^. The PI3K-mTOR pathway is critical in the regulation of cell growth, apoptosis, differentiation, and migration and has been extensively studied in many solid tumors, including PanNETs. In addition to the loss of function mutations, half of the PanNET patients exhibit decreased PTEN expression and 35% with downregulation of TSC2, which significantly correlates with poor survival^34^.

Here, we developed and characterized a novel PanNET mouse model based on the combined deletion of *Men1*, *Atrx*, and *Pten* tumor suppressors.

## Results

**Figure 1:**
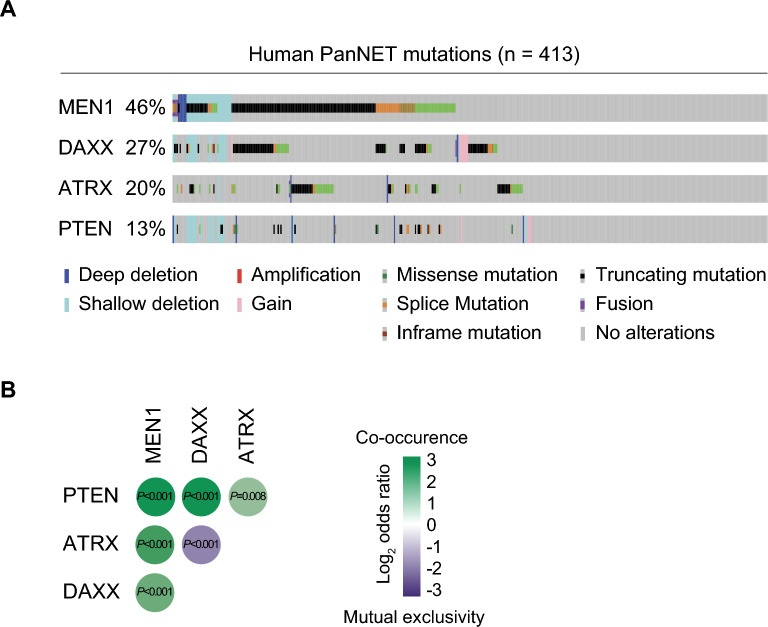
Mutations of MEN1, ATRX, and PTEN co-occur in human PanNETs. (**A**) *MEN1, ATRX,* and *PTEN* tumor suppressor genes are frequently mutated and co-occur in human PanNETs. Oncoprint plot based on AACR GENIE Cohort v14.1 dataset (n = 413 patients). (**B**) Mutual exclusivity and co-occurrence of mutations in human PanNETs. In statistical analyses of the co-occurrence and exclusivity of the most common mutations found in PanNETs, the most notable findings were: (1) the mutual exclusivity between *ATRX* and *DAXX*, which encode proteins forming one functional complex, and (2) the statistically significant co-occurrence of *MEN1, ATRX/DAXX* and *PTEN* mutations in PanNET patients. For odds ratio calculations (see Methods)^35^. *P* values calculated by Fisher's exact test.

**Figure 2:**
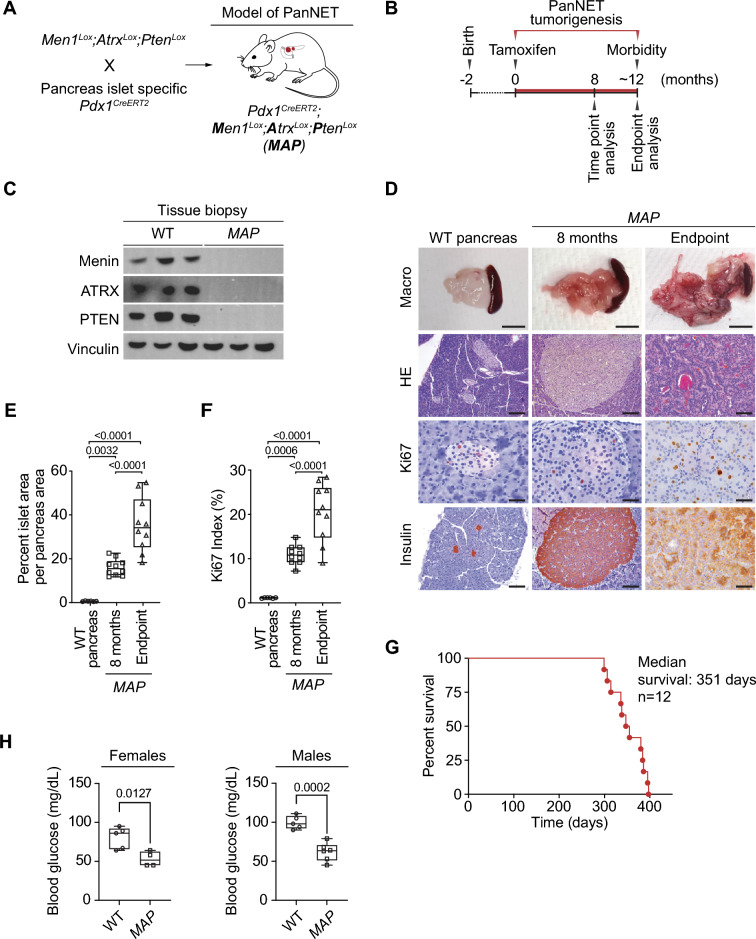
Combined deletion of *Men1, Atrx,* and *Pten* in pancreatic islet cells triggers the development of pancreatic neuroendocrine tumors in mutant mice. (**A**) Schematic illustrating conditional mutant alleles utilized in the generation of PanNET mouse model. To assess the effects of combined *Men1*^*LoxP/LoxP*^, *Atrx *^*LoxP/LoxP*^, and *Pten *^*LoxP/LoxP*^ (*MAP*) ablation on neoplastic transformation of the pancreatic neuroendocrine cells. Tamoxifen-induced recombination of mutant alleles is mediated using pancreatic neuroendocrine cells-specific *Pdx1-CreER* strain. (**B**) Experimental design to investigate tumorigenesis in the *MAP* model following tamoxifen-induced mutant gene recombination (three intraperitoneal injections of 1 mg tamoxifen per mouse every other day) at 8 months and endpoint. (**C**) Representative macroscopic pathology, HE-stained sections and immunohistochemical (IHC) staining with indicated antibodies of pancreas tissue from normal (wildtype) and *MAP* mouse model at 8 months and endpoint. IHC stainings for insulin and Ki67 (a marker of proliferating cells) are shown. Scale bars, 1 cm (macroscopic), 200 µm (histology), 50 µm (Ki67 IHC), representative of n = 10 mice for each experimental group. (**D**) Quantification of neuroendocrine area per total pancreas area in wildtype and *MAP* mutant mice at indicated time points reveals significant expansion of neuroendocrine compartment. *P*-values were calculated by one-way ANOVA with Tukey’s multiple comparisons test. (**E**) Analysis of Ki67 index in normal pancreatic islets and *MAP* mouse model tumors at 8 months and endpoint, revealing a significant increase in tumor cell proliferation. *P* values were calculated by one-way ANOVA with Tukey’s multiple comparisons test. (**F**) Survival analysis of *MAP* mutant mice (n = 10, median survival of 363 days; see Supplementary Fig. 3A for detailed analysis of the clinical spectrum of disease observed in *MAP* mutant model). (**G**) Immunoblotting analysis with indicated antibodies of representative protein lysates of tissue biopsies from wildtype pancreas and *MAP* model tumors. Vinculin is shown as a loading control. (**H**) Fasting blood glucose levels in *MAP* mutant and wildtype (WT) control mice (males and females) at 8 months of age.* P* values were calculated by Student’s *t* test.

To identify key genetic alternations in human PanNETs, we analyzed the genomic sequencing database (GENIE v14.1)^35^. We found that mutations in tumor suppressors *MEN1, ATRX, DAXX*, and *PTEN* are the most frequent (Fig. 1A). Consistent with ATRX and DAXX forming one functional protein complex^36^, mutations in encoding genes are found mutually exclusive in PanNET samples (Fig. 1B). Next, we found that mutations in MEN1, PTEN and ATRX or DAXX co-occur with a high statistical likelihood, suggesting that co-mutation of those genes is one of the core determinants of pancreatic neuroendocrine tumors pathogenesis (Fig. 1B). To explore the tumorigenic potential of the combined loss of MEN1, ATRX, and PTEN in vivo, we interbred conditional *Men1, Atrx* and *Pten* knockout strains with pancreas-specific *Pdx1-Cre*^*ER*^ strain (Fig. 2A). The resulting ***Men1***^LoxP/LoxP^; ***Atrx***^LoxP/LoxP^; ***Pten***^LoxP/LoxP^; *Pdx1-Cre*^*ER*^ mutant mice (hereafter termed ***MAP***) were confirmed by PCR to carry the mutated alleles (Supplementary Fig. 1). PDX1 is expressed in pancreatic multipotent progenitor cells during embryonic development^37,38^, but is restricted to neuroendocrine cells in adult tissue^39^. To remain consistent with the onset of human PanNET in adults and avoid potential embryonic phenotype, we performed tamoxifen injections to induce Cre-mediated recombination in *MAP* mice at 2 months of age (Fig. 2B). Successful depletion of MENIN, ATRX and PTEN was confirmed in tissue lysates (Fig. 2C). Next, *MAP* mutant animals were aged and analyzed at 8 months post tumor induction and at humane endpoint when the animals become moribund. Based on gross pathology examination, all analyzed *MAP* animals developed macroscopic multifocal tumors of variable size in the pancreas (Fig. 2D). Further histological evaluation revealed a significant tenfold increase in pancreatic neuroendocrine cell area in the *MAP* model when compared to wildtype controls at 8 months and progressively larger tumors were observed at endpoint (Fig. 2D, E). To systematically assess PanNETs pathogenesis, we applied the WHO grade system based on the proliferative index (percent of Ki67 positive cells), which clinically serves as a robust prognostic factor in the multivariate analyses and independent predictor for patients survival outcomes^40,41^. The median Ki67 index of *MAP* tumors ranged from 7.2 to 14.8% (mean 10.9%) at the 8-month time point (Fig. 2F), which is consistent with intermediate grade 2 (G2) neuroendocrine tumors (Ki67 index of 3–20%). Of note, we performed similar tumor burden and proliferation analyses of various combinations of *Men1, Atrx,* and *Pten* double-knockouts (Supplementary Fig. 2). Our results indicate that combinations of *Men1;Pten* or *Men1;Atrx* deletion lead to the development of relatively benign well-differentiated grade 1 (G1) tumors (Ki67 index below < 3%), whereas *Atrx;Pten* does not exhibit signs of neoplastic transformation at 8 months post-induction (Supplementary Fig. 2A–C). Overall, these data support the notion that a combined loss of MEN1, ATRX, and PTEN represents an important step in PanNET pathogenesis.

Next, we performed a survival study and found that *MAP* mice develop signs of morbidity and become moribund at approximately 12 months post-tumor induction (median survival of 351 days) (Fig. 2G). Analysis of tumors collected at the endpoint revealed further advancement of PanNET malignancy with a robust over twofold increase in tumor burden and approximately twofold increase in Ki67 index (mean 19.5%) compared to tumors collected at 8 months post-induction (Fig. 2D, E). Notably, 50% of mice at the endpoint developed high-grade tumors (G3) with a Ki67 index > 20% (Fig. 2F), indicative of progressive disease malignancy. However, we have not observed the formation of macroscopic metastasis at the endpoint analysis. Consistent with *Pdx1-Cre*^*ER*^ expression in pancreatic β-cells, the *MAP* model developed PanNET insulinomas (see insulin expression (Fig. 2D). Expectedly, the advancing tumor burden was accompanied by the development of hypoglycemia as monitored by fasting blood glucose measurements (Fig. 2H). Congruent with prolonged hypoglycemia, we observed the deterioration of overall animal health, leading to moribund condition, including limb weakness, partial paralysis or tremors of the hindquarters, and weight loss (Supplementary Fig. 3A-B). In addition, in 1 out of 12 animals, we observed jaundice, which resulted in moribund condition. Together, these in vivo data support a key enabling function for a combined loss of MEN1, ATRX, and PTEN in the initiation and progression of PanNET.

**Figure 3:**
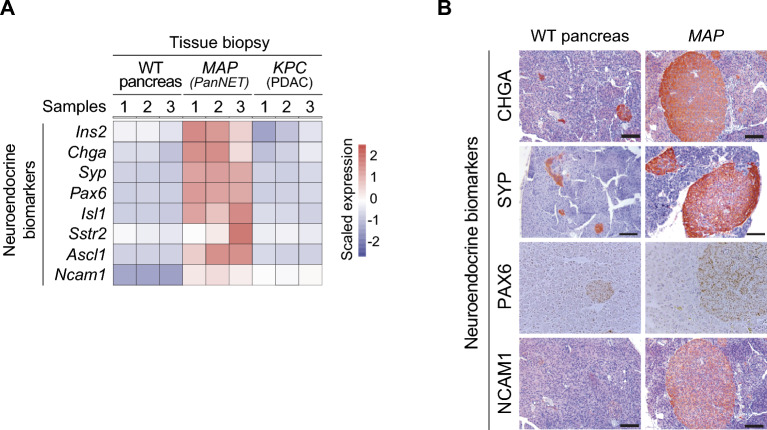
Evaluation of neuroendocrine differentiation and PanNET markers expression in *MAP* mouse model tumors. (**A**) Heatmap of neuroendocrine differentiation and PanNET marker genes expression in RNA-seq datasets obtained from wildtype pancreas (n = 3), *MAP* model of PanNETs (n = 3), and *KPC* model of pancreatic ductal adenocarcinoma (PDAC, n = 3). (**B**) Representative immunohistochemistry images for Chromogranin A (CHGA), Synaptophysin (SYP), PAX6 and NCAM1 staining in wildtype pancreas and *MAP* tumors collected at 8 months. Scale bars, 200 µm.

To further characterize the tumors developed in *MAP* mutant mice, we performed RNA sequencing on micro-dissected tumor biopsies from three independent animals. We observed robustly increased expression of neuroendocrine and PanNET marker genes, including Insulin, Chromogranin A, Synaptophysin, PAX6, ISL1, SSTR2, ASCL1, and NCAM1, in *MAP* tumors compared to normal wildtype pancreas or pancreatic ductal adenocarcinoma biopsies from*** Kras***^*G12D*^*; p53*^*Lox*^*; Ptf1-Cre* (***KPC***) mouse model (Fig. 3A). We further validated the expression of Chromogranin A, Synaptophysin, PAX6 and NCAM1 using immunohistochemistry staining on *MAP* model tumor sections (Fig. 3B). Elevated expression of these markers is consistent and diagnostic for human PanNETs^42,43^, thus indicating the *MAP* model recapitulates biology of pancreatic neuroendocrine malignancy.

**Figure 4:**
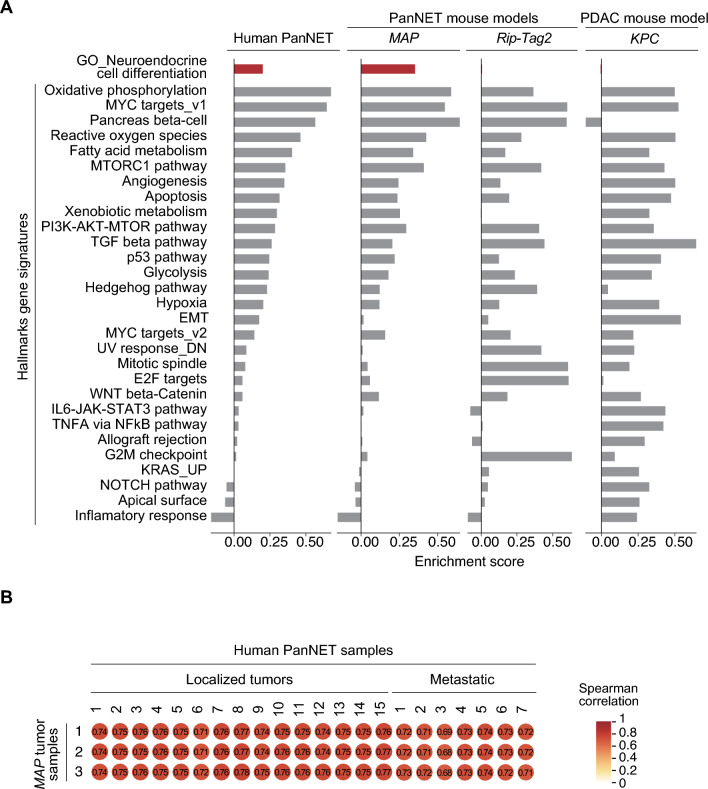
Transcriptional programs observed in *MAP* mouse model tumors are congruent with human PanNETs. (**A**) Single-sample gene set enrichment analysis (ssGSEA) of RNA-seq datasets of PanNETs obtained from patient samples (n = 22), *MAP* (n = 3)*, RIP-Tag2* (n = 31) and pancreatic ductal adenocarcinoma (PDAC) model *KPC* (n = 3). ssGSEA was performed using cancer hallmarks and neuroendocrine cell differentiation signature (GO0061101) from the MSigDB Molecular Signatures Database. RNA-Seq datasets of human PanNETs^44^ and RIP-Tag2 mouse model^45^ were previously published. (**B**) Spearman correlation analysis of transcription profiles between the human PanNET patient samples and MAP mouse PanNET tissues and. The values indicate Spearman correlation coefficient between variables.

Next, we utilized RNA-seq data in single-sample gene set enrichment analysis (ssGSEA) to gain insights into molecular mechanisms involved in *MAP* model tumorigenesis. The score derived from ssGSEA reflects the degree to which specific hallmark gene signatures and pathways are coordinately up or downregulated in analyzed samples. To provide relevant comparison groups, we utilized the same method to analyze RNA-seq datasets of tumor samples from: (1) human PanNETs^44^, (2) *RIP-Tag2* PanNET mouse model^45^, and (3) *KPC* PDAC mouse model. Our computational analyses revealed a high level of agreement between transcriptional signatures found in human PanNETs and *MAP* model tumors, in particular, enrichment in oxidative phosphorylation, reactive oxygen species, fatty acid and xenobiotic metabolism, angiogenesis, MYC and MTOR pathways (Fig. 4A). Notably, *MAP* enriched molecular programs are consistent with human PanNET biology^14–16^. In contrast, we noted that tumors from *RIP-Tag2* mutant mice, a commonly studied PanNET model, are characterized by transcriptional signatures distinct from human and mouse *MAP* tumors, for instance, different enrichment in pathways involved in cell proliferation (G2M checkpoint, mitotic spindle, E2F targets), xenobiotic metabolism and IL6-JAK-IL6-JAK-STAT3 signaling. As expected, human PanNETs and *MAP* model tumors exhibit distinct transcriptional programs from those found in the KRAS-driven pancreatic adenocarcinoma *KPC* model, including those associated with inflammatory response, allograft rejection, NFkB, STAT3, and KRAS pathways. The most characteristic of human and *MAP* PanNETs is the enrichment of neuroendocrine cell differentiation gene signature, which was absent in *RIP-Tag2* and *KPC* models (Fig. 4A).

Finally, we sought to investigate similarities in overall gene expression profiles of *MAP* tumors and PanNET samples representing localized and metastatic disease^44^. Spearman's correlation analysis revealed the statistically significant (*p* < 0.01) strong positive correlation (Spearman’s correlation coefficient > 0.7) between *MAP* and human PanNET transcriptomes (Fig. 4B). Altogether, molecular characterization indicates that MAP model tumors resembled molecular features of human PanNETs.

## Discussion

Pancreatic neuroendocrine tumors are a rare and clinically challenging group characterized by high phenotypic and molecular heterogeneity. Although standard-of-care surgical resection^8^ and chemotherapy^9,10^ improved the prognosis of low-grade PanNETs, high-grade tumors are highly lethal. Therefore, efforts to decipher molecular mechanisms of PanNET pathogenesis are critical in developing novel therapeutic modalities. However, the lack of cellular and animal models of PanNETs hinders research progress. Currently utilized animal models have not faithfully recapitulated human PanNET disease as current models yield mixed tumor types (e.g. pancreatic and pituitary neuroendocrine tumors)^15,46–48^ which have severe phenotypes that confound preclinical findings^49^, or utilize mutations that are not common in PanNETs^47,50,51^. The latter includes RIP-Tag transgenic mice the most commonly used PanNET models, based on the expression of SV40 T antigen, which blocks p53 and Rb family tumor suppressors, mutations rarely found in PanNETs. In this context, sequencing efforts within the last decade have revealed the genetic mutation profile for this disease. These results have shown that loss of *MEN1, DAXX, ATRX,* and genes in PI3K/mTOR pathway (in particular *PTEN* and TSC2 tumor suppressors) are most frequently associated with PanNET tumorigenesis and predict poor prognosis^14–16,52,53^. Further, our analysis shows that *MEN1, DAXX or ATRX* and PTEN mutations commonly co-occur, suggesting that combined loss of those tumor suppressors is likely a key event in pancreatic neuroendocrine cell tumorigenesis. However, we do not have a genetically defined animal model that recapitulates the full spectrum of mutations co-occurring in human PanNETs. Since genetically engineered mouse models of cancer are instrumental in understanding the mechanisms of tumor progression, we sought to establish a model based on the most common and co-occurring PanNET mutations. Our results demonstrated that the combined loss of *Men1*, *Atrx*, and *Pten*, tumor suppressors, triggers the initiation and progression of pancreatic neuroendocrine cell tumorigenesis. The histopathological evaluation of *MAP* tumors at various time points indicates the development of progressive disease, which resembles intermediate to high-grade human PanNETs^54^. In contrast, previous models based on ablation of MEN1 or a combination of MEN1 and PTEN or DAXX deletion yield only low-grade tumors that rarely show signs of disease progression^30,32,48^. Further, our comparative transcriptomic analyses revealed that *MAP* model tumors recapitulate the expression of biomarkers diagnostic for human PanNET including Chromogranin A, Synaptophysin, PAX6 and NCAM1 (Fig. 3)^42,43^. In addition, *MAP* model tumors closely mimic gene expression programs found in human disease (Fig. 4), unlike commonly utilized *RIP-Tag* model exhibiting distinct transcriptomic programs. Altogether, we present here a novel genetically defined animal model of PanNETs that recapitulates human disease at phenotypic, genetic, and transcriptomic levels allowing further application in investigating PanNET biology and therapeutic response.

## Materials and methods

### Animal models

Conditional knockout strains *Men1*^LoxP/LoxP^, *Pten*^LoxP/LoxP^ and *Atrx*^LoxP/LoxP^ have been previously described^55–57^. Tissue-specific recombination of conditional alleles was achieved by interbreeding the conditional knockout alleles with Cre strain expressed from pancreas neuroendocrine cell specific promoter *Pdx1-Cre*^*ER*^ obtained from Jackson Laboratory (#024968) and previously described^58^. The genotype of mice was determined by PCR on DNA obtained from tail snips of new weanlings using specific primers: (a) *Pdx1*-Cre^ER^-F: 5′ACCAGCCAGCTATCAACTCG3′, *Pdx1*-Cre^ER^-R: 5′TTACATTGGTCCAGCCACC, Control-F: -5′CTAGGCCACAGAATTGAAAGATCT3′, Control-R: 5′GTAGGTGGAAATTCTAGCATCATCC; *Men1*-WT-F: 5′ATTGAATAGCCAGAGGGATCTG, *Men1*-WT-R: 5′AGATGCTTGCTCAGTACATTGC, *Men1*-Lox-F: 5′GCCATTTCATTACCTCTTTCTCC; *Atrx*-WT-F: 5′TCAACTGCCCTACATACTGGTG, *Atrx*-Neo-R: 5′CGTGATATTGCTGAAGAGCTTG, *Atrx*-WT-R: 5′GCACGCAAGATAAGAGTGTCTG; *Pten*-F: 5′CAAGCACTCTGCGAACTGAG, *Pten*-R: 5′AAGTTTTTGAAGGCAAGATGC. To induce Cre-recombination and initiate PanNET tumorigenesis *Pdx1*-Cre^ER^; *Men1 *^LoxP/LoxP^; *Atrx* ^LoxP/LoxP^; *Pten*^LoxP/LoxP^ (*MAP*) mutant mice were injected intraperitoneally with 1 mg tamoxifen (Sigma-Aldrich) diluted in 100 μL sunflower oil at 8 weeks of age. For tumor development studies, animals were sacrificed at 8 months after tamoxifen injection and tissues were processed for histological and immunohistochemical analysis. For survival studies, mutant mice were continuously monitored for signs of disease progression and moribund conditions development. At the endpoint, tumors were processed for histological and immunohistochemical analysis. For blood glucose measurement MAP mutant mice at 8 months post-tumor induction and age- and sex-matched wildtype mice were fasted for 4 h before measurement. Blood glucose was measured with ONE TOUCH Ultra2 blood glucose meter (Lifescan, Inc., USA) according to the manufacturer’s instructions. In all experiments, all animals were numbered, and experiments were conducted in a blinded fashion. After data collection, genotypes were revealed, and animals assigned to groups for analysis. None of the mice with the appropriate genotype were excluded from this study or used in any other experiments. All mice were co-housed with littermates (2–5 per cage) in pathogen-free facility with standard controlled temperature of 72 °F, with a humidity of 30–70%, and a light cycle of 12 h on/12 h off set from 7 a.m. to 7 p.m. and with unrestricted access to standard food and water under the supervision of veterinarians, in an AALAC-accredited animal facility at the University of Texas M.D. Anderson Cancer Center (MDACC). Mouse handling and care followed the NIH Guide for Care and Use of Laboratory Animals. All animal procedures followed the guidelines of and were approved by the MDACC Institutional Animal Care and Use Committee (IACUC protocol 00001636, PI: Mazur).

### Histology and immunohistochemistry

Mice were euthanized by CO_2_ asphyxiation followed by cervical dislocation at the indicated time points or when moribund conditions developed (endpoint). Tissue specimens were then fixed in 4% zinc-buffered formalin for 48 h and stored in 70% ethanol until processing and paraffin embedding. 1.5 μm sections were prepared and stained with Hematoxylin and Eosin (HE) or used for immunohistochemical studies (IHC). IHC was performed on formalin-fixed, paraffin-embedded mouse tissue section using a biotin-avidin method as previously described^59^. The following antibodies were used (at the indicated dilutions): insulin (CST #3014, 1:2000), synaptophysin (Abcam #ab32127, 1:1000), Ki67 (CST #12202, 1:800), CD56/NCAM (Proteintech #14255-1-AP, 1:2000) and ChgA (Abcam #ab15160, 1:1000). Sections were developed with DAB and counterstained with hematoxylin. Pictures were taken using a PreciPoint M8 microscope equipped with PointView software. The percentage of islet area or tumor area per pancreas was quantified based on HE staining utilizing Image J software. Cell proliferation was quantified using Ki67 marker. Ki67-positive and negative cells were quantified for each tumor field at 400 × magnification (5 representative tumor fields per tumor sample were analyzed). The Ki67 or mitotic index was calculated as the percentage of positively stained cells per the total number of cancer cells assessed.

### Immunoblotting analysis

For immunoblotting analysis, wildtype pancreas and *MAP* model tumor biopsies were collected and homogenized in RIPA buffer with 1 mM PMSF and a complete protease inhibitor cocktail (Roche) to prepare whole-cell protein lysates. Protein concentration was determined using the BCA protein assay kit (Pierce #23227). Protein samples were resolved by SDS-PAGE and transferred to a PVDF membrane (0.45 µm). The following antibodies were used (at the indicated dilutions): MEN1 (1:1000, CST #19893), PTEN (1:1,000, CST #9188), ATRX (1:1000, CST #10321), Vinculin (1:10,000, CST #13901) and HRP-conjugated secondary antibody (1:10,000, CST #7074). Protein bands were visualized using Amersham ECL or Amersham ECL Prime western blotting detection reagent.

### RNA isolation and sequencing

RNA samples were extracted from fresh tumor biopsies from the MAP model and pancreatic ductal adenocarcinoma model: *Kras*^*G12D*^*; p53*^*Lox/lox*^*; Ptf1a-Cre* (KPC) previously described^60^. Briefly, biopsied tumors were homogenized with TRIzol reagent (Life Technologies) and centrifuged at 16,000 RPM for 15 min at 4 °C. The samples received additional precipitation with a 1:2 chloroform:isoamyl alcohol pH 8.0 solution and centrifuged at 12,000 RPM for 15 min to promote phase separation of RNA. Three layers formed from this precipitation: an upper aqueous phase (containing RNA), a white interphase (containing DNA), and a lower organic phenol:chloroform phase (consisting of proteins). The upper aqueous phase was collected and mixed 1:1 with 100% ethanol. The Direct-zol RNA miniprep kit (cat. no. R2050) was utilized. RNA samples were sequenced at 20 M reads, PE150 (Novogene).

### Human and mouse PanNET RNA-seq analyses

Previously reported RNA-seq datasets of human PanNETs^44^ were obtained from Gene Expression Omnibus (accession no. GSE178398) and *RIP-Tag2* mouse model^45^ from the ZENODO database (access: doi.org/10.5281/zenodo.4160441). RNA-seq data of *MAP* and *KPC* models were generated in-house and were mapped, counted, and normalized to TPM. Shared gene homologs between the human and mouse samples were kept for downstream processing. Correlation analysis was performed in R, and the results were plotted using the R package *Corrplot.*

### Single-sample gene set enrichment analysis (ssGSEA)

Single-sample gene set enrichment analysis (ssGSEA) for each pancreatic tumor sample was performed using the R package *GSVA* on cancer hallmarks^61^ and GO neuroendocrine gene set (GO0061101) from the MSigDB Molecular Signatures Database.

### Human PanNET mutation analysis

To identify genetic alternations critical for PanNET development, we performed mutation analysis of recurring mutations in human PanNETs using AACR GENIE Cohort v14.1 dataset (n = 413 patients) from cBioPortal v5.3.13^35,62^. To identify patterns of mutual exclusivity or co-occurrence, we performed statistical analysis for each pair of queried genes (*MEN1, ATRX, DAXX* and *PTEN*) that computes the likelihood that the mutations in each pair of genes are mutually exclusive or co-occurrent across the selected PanNET patient samples. The odds ratio (OR) is calculated with formula: OR = (A * D)/(B * C), where A = number of cases altered in both genes; B = number of cases altered in gene 1 but not gene 2; C = number of cases altered in gene 2 but not gene 1; and D = number of cases altered in neither genes.

### Ethics approval

All animal experiments in this study were approved by the MDACC Institutional Animal Care and Use Committee (IACUC protocol 00001636, PI: Mazur) and conducted in compliance with the ARRIVE guidelines.

### Supplementary Information

Supplementary Information.

## Data Availability

The RNA-seq data that support the findings of this study have been deposited in the Gene Expression Omnibus (GEO) database under accession number GSE248606. Review access tokens: gnavgwiwvtahfyx.
