# Supplementary material for: Combined deletion of MEN1, ATRX and PTEN triggers development of high-grade pancreatic neuroendocrine tumors in mice
Source: Sci Rep. 2024 Apr 12;14:8510. doi: 10.1038/s41598-024-58874-2 (PMC11014914; doi:10.1038/s41598-024-58874-2)
Supplement: Supplementary file 1 — Supplementary Information. [file 41598_2024_58874_MOESM1_ESM.pdf]

Supplementary Figure 1

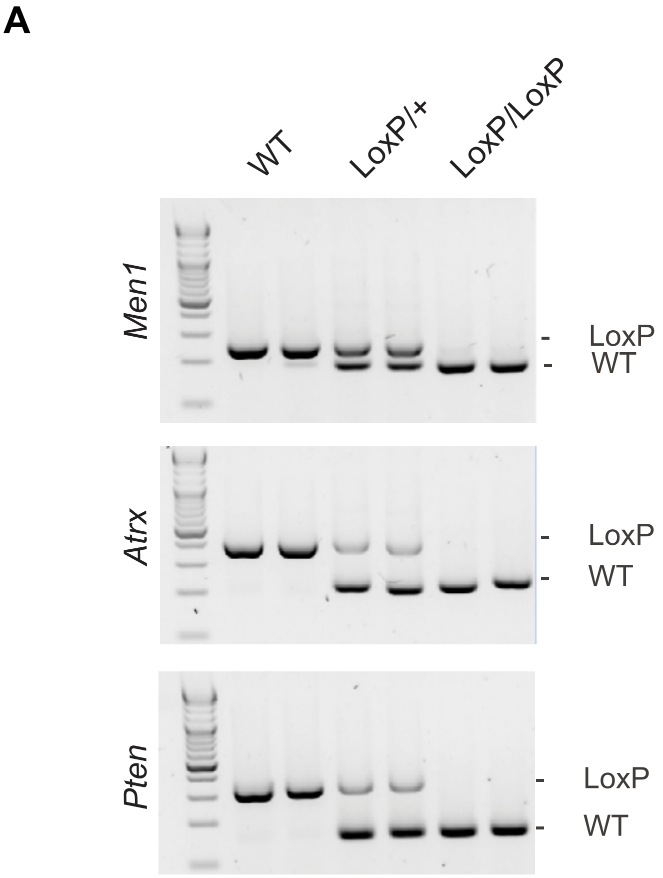

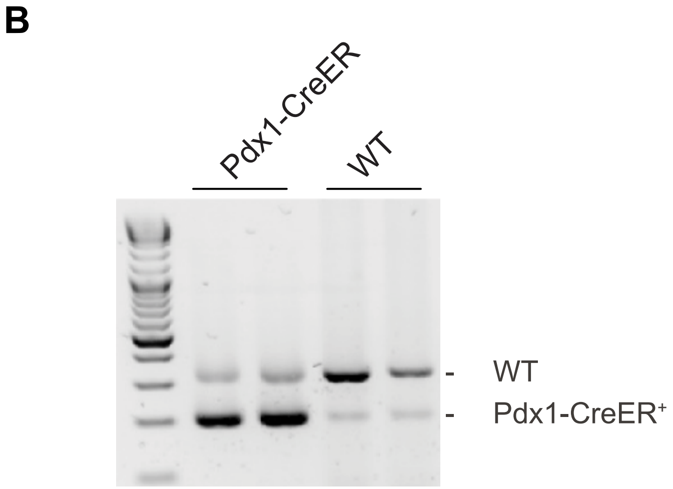

Supplementary Figure 1. Confirmation of *Pdx1-CreER*, *Men1*$^{LoxP/LoxP}$, *Atrx*$^{LoxP/LoxP}$, and *Pten*$^{LoxP/LoxP}$ mutant mouse strains.

A-B. Confirmation of *Men1*$^{LoxP/LoxP}$, *Atrx*$^{LoxP/LoxP}$, and *Pten*$^{LoxP/LoxP}$ conditional alleles (A) and *Pdx1-CreER* transgenic strain (B) by PCR on DNA isolated from mouse tail biopsies from indicated mouse genotypes, expected products sizes are marked (see Methods).

Supplementary Figure 2

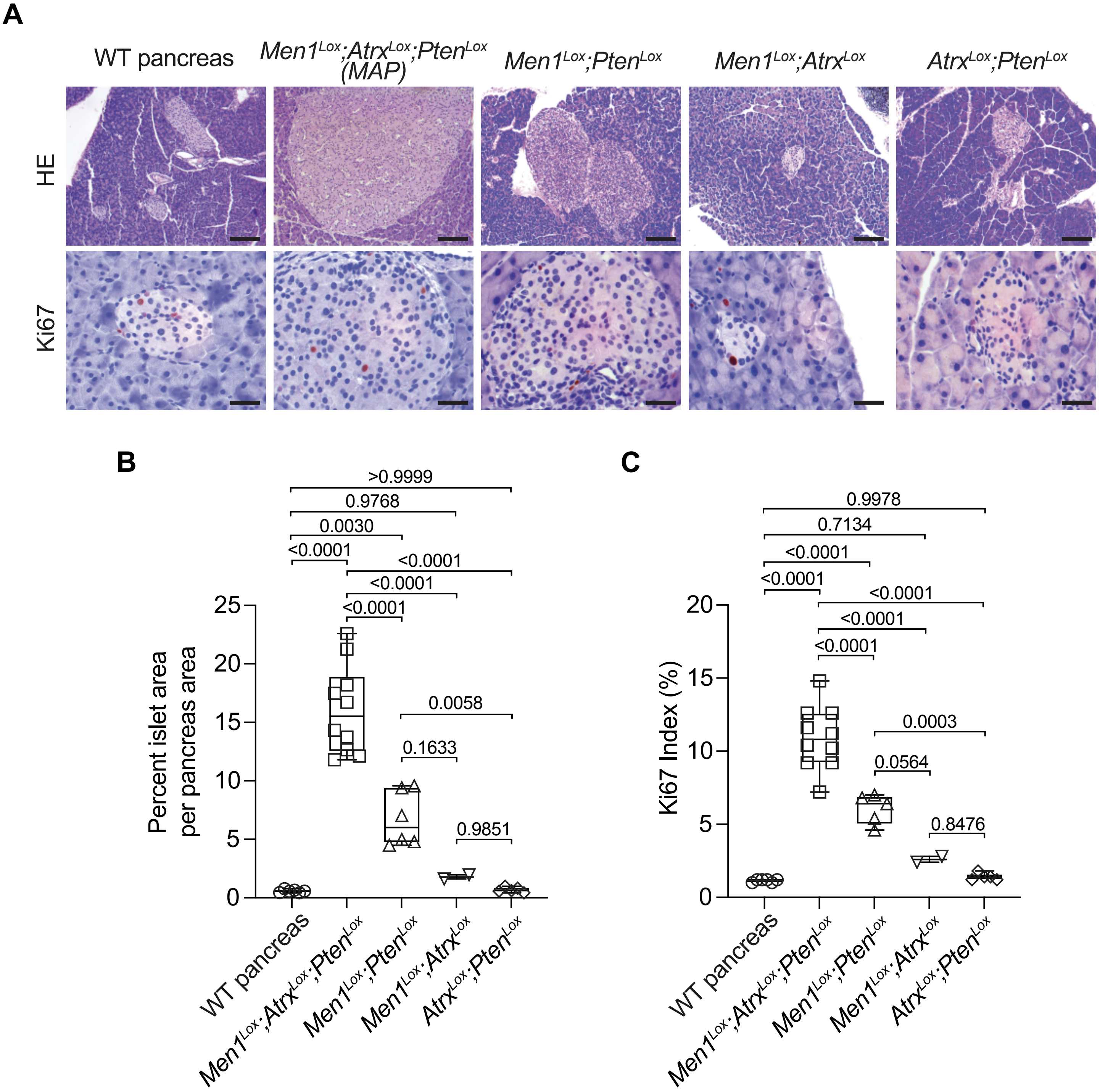

Supplementary Figure 2. Analyses of tumorigenic potential of pancreas neuroendocrine specific co-deletions of *Men1*$^{LoxP/LoxP}$, *Atrx*$^{LoxP/LoxP}$, and *Pten*$^{LoxP/LoxP}$.

A. Representative HE staining and Ki67 immunohistochemistry (marker of proliferating cells) of pancreas tissue from wildtype and indicated mutant strains. Tamoxifen-induced recombination of indicated conditional mutant alleles is mediated using pancreatic neuroendocrine cells-specific *Pdx1-CreER* strain. Analyses performed at 8 months after tumor induction (as in Fig 2B). Scale bars, 200 µm (histology) and 50 µm (Ki67 IHC).

B. Analyses of pancreatic neuroendocrine cells area per total pancreas area in wildtype, and indicated mutant strains at 8 months after tumor induction. *P*-values were calculated by one-way ANOVA with Tukey's multiple comparisons test.

C. Quantification of Ki67 index of pancreatic neuroendocrine cells from wildtype and indicated mutant strains at 8 months after tumor induction. *P*-values were calculated by one-way ANOVA with Tukey's multiple comparisons test.

Supplementary Figure 3

A

Mouse ID	Sex	Survival (days)	Observations	Insulin	NET marker Synaptophysin	Tumor burden	Ki67 Index (%)
1	F	338	WL	+	+	18.3	15.4
2	F	386	WL, LW	+	+	28.8	10.4
3	F	306	WL, LW	+	+	26.4	11.2
4*	F	314	WL, LW	n.a.	n.a.	n.a.	n.a.
5*	F	355	WL, J	n.a.	n.a.	n.a.	n.a.
6	M	384	WL, LW	+	+	21.8	12.4
7	M	397	WL, PPT	+	+	19.5	15.8
8	M	347	WL, LW	+	+	30.7	20.4
9	M	380	WL, PPT	+	+	45.1	25.2
10	M	336	WL, LW	+	+	32.5	19.6
11	M	299	WL, LW	+	+	53.2	25.4
12	M	395	WL, LW	+	+	54.7	28.4

WL, Weight loss
LW, Limb weakness
PPT, partial paralysis/tremors of the hindquarters
J, Jaundice
n.a., not analized
*, post mortem

B

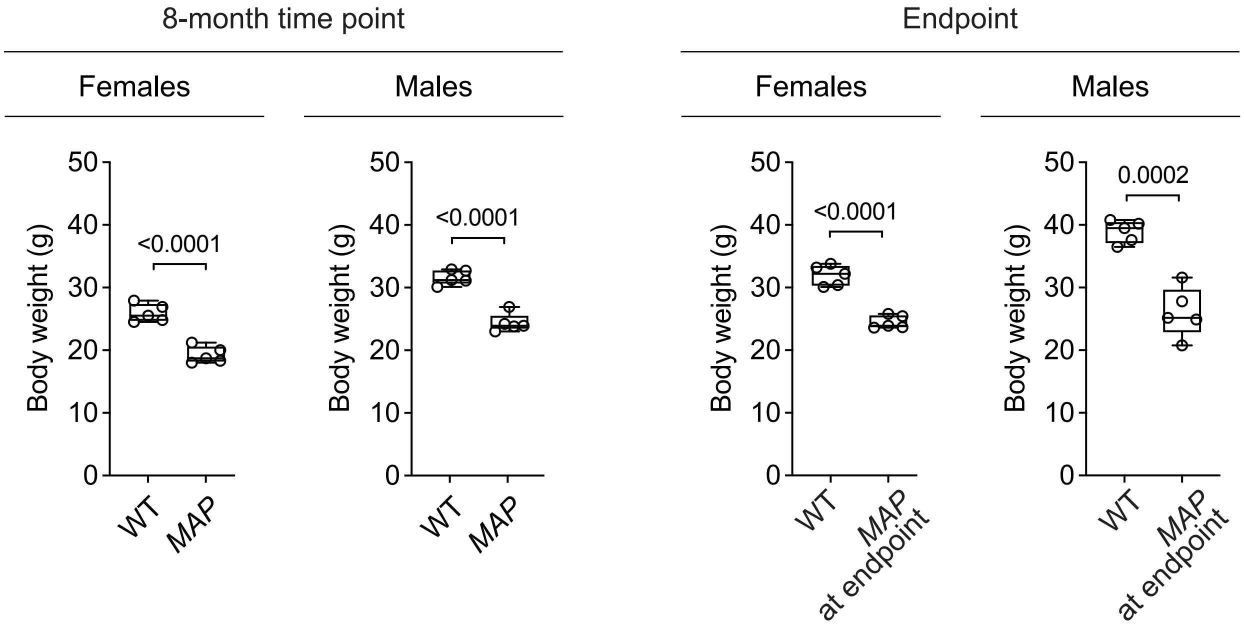

Supplementary Figure 3. Evaluation of clinical spectrum of disease and expression of neuroendocrine differentiation markers in *MAP* mouse model

A. Analysis of the clinical spectrum of disease observed in *MAP* mutant model at endpoint.

B. Body weight of *MAP* mutant mice at 8-month timepoint and endpoint. Age-matched wildtype mice are shown as control. *P*-values were calculated by the Student's t-test.

Supplementary Figure 4

Uncproped immunoblot images - Figure 2C

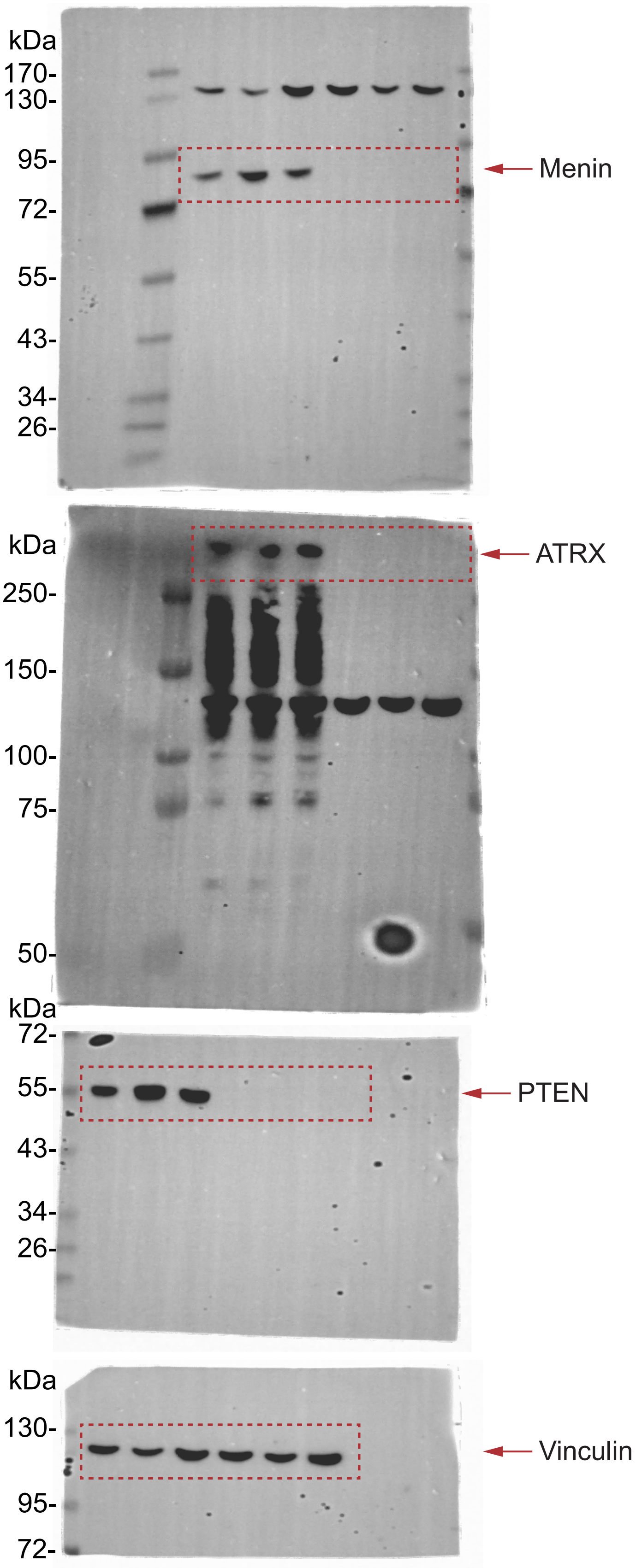

Supplementary Figure 4. Source data - uncropped immunoblotting images from Figure 2C.
